# Early childhood internalizing problems, externalizing problems and their co-occurrence and (mal)adaptive functioning in emerging adulthood: a 16-year follow-up study

**DOI:** 10.1007/s00127-020-01959-w

**Published:** 2020-09-22

**Authors:** İldeniz B. Arslan, Nicole Lucassen, Pol A. C. van Lier, Amaranta D. de Haan, Peter Prinzie

**Affiliations:** 1 Department of Psychology, Education and Child Studies, Erasmus University Rotterdam, Burgemeester Oudlaan 50, 3000 Rotterdam, The Netherlands; 2 Department of Clinical, Neuro and Developmental Psychology, VU University Amsterdam, De Boelelaan 1105, 1081 Amsterdam, The Netherlands

**Keywords:** Early childhood, Internalizing and externalizing problems, Co-occurrence, (Mal)Adaptive functioning, Emerging adulthood

## Abstract

**Purpose:**

A vast amount of studies suggest that internalizing or externalizing problems are related to individual functioning, and often co-occur. Yet, a focus on their additive and interactive effects is scarce. Furthermore, most research has focused on a limited number of developmental domains and mostly on maladaptive functioning. Therefore, the current prospective study examined whether early childhood (ages 4–8) internalizing and externalizing problems and their interaction were related to a broad range of (mal)adaptive functioning outcomes in emerging adulthood (ages 20–24).

**Methods:**

Data from the Flemish Study on Parenting, Personality and Development were used. At Time 1 (1999) mothers of 374 children (45% boys) and fathers of 357 children (46% boys) rated internalizing and externalizing problems through the Child Behavior Checklist. Outcomes in emerging adulthood were measured through self-reports 16 years later across the following domains: psychological functioning, social functioning, work, physical health, and self-concept.

**Results:**

Early externalizing problems were related to maladaptive outcomes on the psychological and social domains. With regard to adaptive functioning, externalizing problems were associated with lower satisfaction regarding general health on the physical domain. Early internalizing problems were not associated with any emerging adulthood outcomes. The interaction of (father reported) internalizing and externalizing problems was related to aggressive behavior.

**Conclusion:**

Early childhood externalizing problems were associated with maladaptive and adaptive functioning over a time span of 16 years. The results add to studies on the implementation of prevention and intervention programs in early childhood and to the value for developing personalized interventions.

**Electronic supplementary material:**

The online version of this article (10.1007/s00127-020-01959-w) contains supplementary material, which is available to authorized users.

## Introduction

“Give me a child until he is seven and I will show you the man” might be one of the best-known quotes of Aristotle, underpinning the theoretical importance and influence of early childhood on long-term outcomes. A vast amount of studies suggest that internalizing and externalizing problems have concurrent and longitudinal effects on individual functioning (e.g., [1–3]) and often co-occur (e.g., [4]). However, these studies face limitations with regard to the generalizability of their findings as a focus on additive and interactive effects of internalizing and externalizing problems in population samples is scarce. Also, as most studies focused on maladaptive societal functioning outcomes, relatively less is known about general functioning in emerging adulthood, in maladaptive and adaptive terms. Furthermore, studies on early childhood functioning are commonly based on single informants. This study adds to current knowledge by examining the extent to which additive and interactive effects of early childhood internalizing and externalizing problems, rated by mothers and replicated by father reports, are related to maladaptive and adaptive general functioning in emerging adulthood.

## Early childhood internalizing and externalizing problems

Externalizing problems entail aggressive and oppositional behavior, whereas internalizing problems consist of anxious and depressed symptoms, withdrawn behavior, and somatic symptoms [5]. Externalizing problems predict a variety of functioning outcomes, such as delinquency throughout adolescence [6], social problems from middle childhood to emerging adulthood [1] and poor academic achievement from middle childhood to early adolescence [7]. Internalizing problems have received relatively less attention. Yet, studies have pointed out that internalizing problems can have negative consequences as well. For example, Goodwin and colleagues [8] reported that childhood internalizing problems were associated with anxiety and depression in adolescence and emerging adulthood. Although often studied as two separate domains, internalizing and externalizing problems often co-occur (e.g., [9]). Co-occurring problems are present in childhood, adolescence, and adulthood. However, the onset of co-occurrence is most often found in early childhood [4, 10]. This emphasizes the importance of studying additive and interactive associations of early childhood internalizing and externalizing problems, particularly in terms of a better understanding of their long-term prospective associations with developmental outcomes [11]. Remarkably, as the literature predominantly consists of studies focusing on internalizing or externalizing problems only, thereby ignoring their co-occurrence (e.g., [12–16], there is little knowledge about the relative impact of one type of problem, while controlling for the other types of problems. A few studies provide knowledge about the role of co-occurring internalizing and externalizing problems on maladaptive emerging adulthood functioning. Sourander and colleagues [17] found that boys who scored high on internalizing and externalizing problems at 8 years old were at risk for psychiatric disorders, criminal offenses, and substance abuse in emerging adulthood. Further, Capaldi and Stoolmiller [18] reported that early adolescent boys with co-occurring problems scored high on maladaptive outcomes, such as driver’s license suspensions and early fatherhood in emerging adulthood.

Multiple methodological issues hamper our knowledge on the wicked problem of studying child internalizing and externalizing problems. Studies vary largely concerning the conceptualization by referring to internalizing and externalizing problems in non-clinical terms (e.g., [4, 7]), or to clinical disorders such as emotional disorders (e.g., [15, 16]). Most studies analyzed internalizing and externalizing problems by creating groups (also called the discrete method) of different types of problems based on (clinical) cutoff scores [1, 8, 13, 14, 17, 19–22]. Internalizing and externalizing problems can also be analyzed as a continuum. This continuous approach is especially beneficial in population studies, as it provides more reliable and valid assessments, taking into account the full range of internalizing and externalizing problems [23, 24]. High rates of problems tend to be less frequent in community samples, thus, a continuous method allows for a better representation of the distribution of *symptoms* [25]. Also, some studies showed that rates of internalizing and externalizing problems below certain thresholds are associated with outcomes comparable to those scoring above these thresholds [26]. As our study is conducted within a community sample, we apply a continuous method in examining internalizing and externalizing problems. This approach of examining internalizing and externalizing problems is similar to the continuous method that was applied in the study by Capaldi and colleagues [18]. They examined additive and interactive associations between early adolescence internalizing and externalizing problems, and emerging adulthood functioning on multiple domains. In a first step, Capaldi and colleagues [18] operationalize internalizing and externalizing problems as two dimensions, and in the second step of their dimensional approach, interaction terms of internalizing and externalizing problems are created. Examining the interactive effects of internalizing and externalizing problems yields important knowledge effects about the question whether internalizing problems depend on levels of externalizing problems, and vice versa. Therefore, in our study, we define co-occurrence in twofold, namely, co-occurrence is represented by (1) main effects of internalizing and externalizing problems, controlled for each other and (2) the interaction term of internalizing and externalizing problems.

## (Mal)Adaptive functioning in emerging adulthood

There is growing evidence that internalizing and externalizing problems can affect several domains of functioning rather than a single domain [22]. Thus, especially to better understand the associations of childhood internalizing and externalizing problems as developmental precursors of functioning in emerging adulthood, it is important to extend the range of outcomes. The concept of “emerging adulthood” as introduced by Arnett [27] refers to a period of transition into adulthood and underpins the importance of giving attention to a wide range of outcomes. As many emerging adults undergo life changes such as leaving their homes, start working, and taking on adult responsibilities, developmental functioning areas such as identity development and functioning with regard to work and romantic relations [27, 28] are important to study. Previous studies on the association of internalizing and externalizing problems focused mainly on maladaptive societal functioning in emerging adulthood, such as criminal behavior [1], substance use [17], mental disorders [1], and more specific outcomes such as early exit from the family-of-origin home [17] and early pregnancy [18]. However, the study of longitudinal relations in population studies to more general development and functioning in emerging adulthood, such as personality, identity, social relations or sleep behavior in population studies is lacking. Personality pathology, for example, is a relatively understudied topic in relation to co-occurrence and also in terms of its longitudinal antecedents. Emerging adulthood is a prominent developmental period in life for personality development and, consequently, personality pathology [29]. In their review, Kongerslev and colleagues [30] urged for more prospective research on childhood psychopathology and relations with personality pathology, as most studies on early indicators of personality pathology focused on adolescence and emerging adulthood onwards. The concept “emerging adulthood” refers to the age of identity exploration, where self-focus plays an eminent role [28]; hence, self-concept outcomes such as self-efficacy and identity development are important markers of emerging adulthood functioning as well [31]. Knowledge of additive and interactive effects of childhood internalizing and externalizing problems as antecedents of self-concept outcomes in emerging adulthood is sparse. Therefore, the current study applies a multi-faceted approach of emerging adulthood functioning with an emphasis on general functioning. In doing so, it is essential to also pay attention to indicators of (impeded) healthy general functioning besides maladaptive functioning, as it is an important marker for a better quality of life [32]. Surprisingly, the focus on maladaptive functioning characterizes most developmental psychology research [33]; thus, healthy/adaptive functioning remains an understudied topic. However, the absence of problems is not equal to adaptive functioning [34]. Hence, the longitudinal associations of early internalizing and externalizing problems cannot be examined by only including maladaptive outcomes and, therefore, in this study the focus will be on maladaptive and adaptive functioning in emerging adulthood.

## Aims and hypotheses

The overall aim of the current study is to increase knowledge about whether early childhood (4–8 years) internalizing, externalizing problems and their interaction are related to general functioning assessed 16 years later in emerging adulthood (20–24 years). In addition to maladaptive functioning, we also focus on adaptive functioning as this is relatively understudied in relation to earlier internalizing and externalizing problems.

Interventions starting in early childhood are found to be more beneficial on the long term, compared to interventions starting in adolescence or adulthood [35]. Identifying associations of early childhood internalizing and externalizing problems with a variety of general functioning outcomes in emerging adulthood can provide new insights for prevention and intervention programs that are implemented at an early age. As early childhood co-occurrence of internalizing and externalizing problems remains a relatively understudied topic, this study contributes to a better understanding of their role as longitudinal indicators of (impeded) general functioning in emerging adulthood. This knowledge on co-occurrence of internalizing and externalizing problems can aid in the development of personalized interventions.

In the current study, it is expected that early internalizing and externalizing problems are related to outcomes across a wide range of domains of general functioning: psychological domain (i.e., anxiety, delinquency, personality pathology), social domain (i.e., social problems, satisfaction regarding friendships), work domain (i.e., satisfaction regarding work), physical domain (i.e., general health satisfaction), and the self-concept domain (i.e., identity, self-efficacy). With regard to the interactive effects, we expect that the longitudinal associations between externalizing problems and emerging adulthood functioning will be stronger for children that show internalizing problems as well (and vice versa).

## Methods

### Participants and procedure

The current study is part of the ongoing longitudinal Flemish Study on Parenting, Personality and Development consisting of nine waves (1999–2018). Data of the first and eighth wave were analyzed as these waves contained the measures of interest and allowed to answer our research aims. The original sample was randomly selected from 167 schools in Belgium (Flanders). Strata were constructed based on geographical location (province), child gender and age resulting into a proportional stratified sample of 800 invited families at Time 1 (T1). Written informed consent was given and confidentiality was guaranteed. All participants had the  Flemish nationality. The study procedures were approved by the board of the Katholieke Universiteit Leuven. A more detailed description of the study design and recruitment of participants is presented previously (see e.g., [36]). The final sample consisted of participants from whom complete information on Time 2 (T2) was available (56.2% and 56.8% of the original sample for mothers and fathers, respectively). Little’s Missing Completely at Random (MCAR) test showed that the data of mothers (*χ*^*2*^(218) = 242.97, *p* = 0.118) and fathers (*χ*^*2*^(199) = 198.76, *p* = 0.491) were missing completely at random. There were no significant differences on early internalizing problems (mother reports T1, *t*(664) = − 0.32, *p* = 0.751; father reports T1, *t*(626) = -0.09, *p* = 0.926), early externalizing problems (mother reports T1, *t*(664) = 1.26, *p* = 0.209; father reports T1, *t*(626) = − 3.17, *p* = 0.751), and child age (mother reports T1, *t*(664) = − 0.95, *p* = 0.341; father reports T1, *t*(626) = − 1.17, *p* = 0.242) between participants who participated at T1 and T2 and participants who participated at T1 only. Only for the mother-reported data, and not father-reported data (father reports T1, *t*(568) = − 1.30, *p* = 0.195), significant differences were found for educational level of both parents, *t*(656) = − 3.54, *p* < 0.001: participants for whom data were available at both waves had higher educated parents (*M* = 2.64, SD = 0.83) than participants who did not participate at T2 (*M* = 2.41, SD = 0.85). The final sample for mother-reported data consisted of 374 children: 170 boys (*M*_age_ at T1 = 5.83 years, range at T1 = 4.00–8.25 years, *M*_age_ at T2 = 21.83 years, SD = 1.10) and 204 girls (*M*_age_ at T1 = 5.89 years, range at T1 = 4.08–8.33 years, *M*_age_ at T2 = 21.89 years, SD = 1.12). The final sample for father-reported data consisted of 357 children: 166 boys (*M*_age_ at T1 = 5.82 years, range at T1 = 4.00–8.25 years, *M*_age_ at T2 = 21.82 years, SD = 1.10) and 191 girls (*M*_age_ at T1 = 5.88 years, range at T1 = 4.08–8.17 years, *M*_age_ at T2 = 21.88 years, SD = 1.12).

### Measures

Table 1 shows Cronbach’s alphas for mother and father data, number of items, and example items of all measures.

**Table 1:** Descriptive statistics of all variables of interest

	Mother data				Father data				*N* items	Example item
	*M*	SD	Range	*α*	*M*	SD	Range	*α*		
Early internalizing problems	5.01	4.69	0.00–28.00	0.82	4.28	4.39	0.00–23.00	0.82	32	Too fearful or anxious
Early externalizing problems	8.54	6.82	0.00–40.00	0.88	7.84	6.64	0.00–37.00	0.89	33	Impulsive or acts without thinking
Psychological domain										
Internalizing dimension										
Anxious/depressed behavior	9.07	6.89	0.00–34.94	0.90	9.05	6.95	0.00–34.94	0.91	18^a^	I feel lonely
Withdrawn behavior	2.60	2.75	0.00–14.00	0.79	2.60	2.79	0.00–14.00	0.71	9	I would rather be alone than with others
Somatic complaints	3.03	2.96	0.00–18.00	0.72	2.93	2.89	0.00–18.00	0.80	12	I feel dizzy or lightheaded
Externalizing dimension										
Aggressive behavior	4.90	3.75	0.00–21.00	0.80	4.90	3.76	0.00–21.00	0.80	15	I get in many fights
Delinquency	2.58	2.70	0.00–18.00	0.69	2.55	2.65	0.00–18.00	0.67	14	I do things that may cause me trouble with the law
Intrusive behavior	2.88	2.36	0.00–11.00	0.75	2.89	2.39	0.00–11.00	0.82	6	I try to get a lot of attention
Other problems										
Thought problems	4.14	2.53	0.00–13.00	0.63	4.09	2.50	0.00–13.00	0.62	10	I have thoughts that other people would think are strange
Attention problems	8.06	4.98	0.00–25.00	0.83	8.04	5.00	0.00–25.00	0.83	15	I am too forgetful
Personality pathology										
Negative affect	1.96	0.44	1.00–3.29	0.91	1.96	0.44	1.00–3.29	0.91	28	I get emotional easily, often for very little reason
Detachment	1.45	0.41	1.00–3.10	0.91	1.44	0.41	1.00–3.10	0.91	20	I am not interested in making friends
Antagonism	1.60	0.42	1.00–3.20	0.91	1.61	0.42	1.00–2.95	0.91	20	I often have to deal with people who are less important than me
Disinhibition	2.02	0.36	1.10–2.95	0.80	2.02	0.36	1.10–2.95	0.80	20	People would describe me as reckless
Psychoticism	1.43	0.42	1.00–2.75	0.86	1.42	0.41	1.00–2.75	0.85	12	I often “zone out” and then suddenly come to and realize that a lot of time has passed
Social domain										
Social problems	3.82	2.76	0.00–14.00	0.67	3.81	2.77	0.00–14.00	0.67	11	I do not get along with other people
Satisfaction friendships	7.91	1.44	1.00–10.00	–	7.91	1.46	1.00–10.00	–	1	How satisfied are you regarding your friendships in general?
Satisfaction romantic relations	6.48	2.83	1.00–10.00	–	6.54	2.79	1.00–10.00	–	1	How satisfied are you regarding your romantic life in general?
Work domain										
Work satisfaction	7.70	1.59	2.00–10.00	–	7.71	1.58	2.00–10.00	–	1	How satisfied are you regarding your current work in general?
Physical domain										
Satisfaction general health	7.41	1.51	1.00–10.00	–	7.41	1.52	1.00–10.00	–	1	How satisfied are you regarding your health in general?
Sleep problems	2.24	0.60	1.00–4.29	0.78	2.24	0.59	1.00–4.29	0.78	7	How many times do you usually wake up during a night’s sleep?
Self-concept domain										
Exploration in breadth	3.69	0.68	1.00–5.00	0.85	3.67	0.69	1.00–3.67	0.85	5	I think a lot about how I see my future
Ruminative exploration	2.76	0.89	1.00–5.00	0.87	2.75	0.90	1.00–2.75	0.88	5	I worry about what I want to do with my future
Identification with commitment	3.41	0.76	1.00–5.00	0.89	3.41	0.77	1.00–3.41	0.89	5	Future plans give me self-confidence
Commitment making	3.59	0.90	1.00–5.00	0.96	3.58	0.91	1.00–3.58	0.96	5	I know what I want to achieve in my life
Exploration in depth	3.56	0.68	1.00–5.00	0.83	3.54	0.69	1.00–3.54	0.83	5	I talk regularly with other people about the plans for the future I have made
Separation from parents	2.21	0.39	1.00–4.00	0.79	2.20	0.39	1.00–2.20	0.79	12	I go to my parents for help before trying to solve a problem myself
Detachment from parents	2.91	0.47	1.00–3.25	0.77	2.90	0.47	1.00–2.90	0.77	8	I wish my parents would understand who I really am
Self-efficacy	2.89	0.40	1.00–4.00	0.84	2.89	0.38	1.00–2.89	0.84	10	Thanks to my resourcefulness, I can handle unforeseen situations

^a^Scores on item “I worry about my relations with the opposite sex” from the anxious/depressed behavior scale were missing

#### Early internalizing and externalizing problems

At T1, children’s internalizing and externalizing problems were assessed using mother reports and father reports of the Child Behavior Checklist (CBCL [5, 37]). Raw sum scores of the broadband scales *internalizing problems* and *externalizing problems* were used. Items were rated on a three-point scale with 0 (*not true*), 1 (*somewhat/sometimes true*), and 2 (*very/often true*).

#### Psychological domain

At T2, the emerging adults rated maladaptive psychological functioning using self-reports. First, psychological problems were assessed using the Adult Self-Report (ASR [38]). The internalizing dimension consisted of anxious/depressed behavior, withdrawn behavior, and somatic symptoms. The externalizing dimension consisted of aggressive behavior, delinquency, and intrusive behavior. Thought problems and attention problems were assessed as well. Items were rated on a three-point scale with 0 (*not true*), 1 (*somewhat/sometimes true*), and 2 (*very/often true*).

Second, personality pathology was assessed using the 100-item shortened Personality Inventory for DSM-5 (PID-5-SF [39]). The PID-5 assesses five pathological personality dimensions: Negative Affect (i.e., emotional lability, separation insecurity), Detachment (i.e., withdrawal, avoiding intimacy), Antagonism (i.e., manipulativeness, grandiosity), Disinhibition (i.e., irresponsibility, impulsivity), and Psychoticism (i.e., unusual beliefs and experiences, eccentricity).

#### Social domain

At T2, maladaptive social functioning (i.e., not getting along with others or being bullied) was measured through the Social problems scale of the Youth Self-Report (YSR [38]). Two questions were asked to asses adaptive social functioning: emerging adults’ satisfaction concerning their friendships and with regard to their romantic relations, both measured on a ten-point scale with 1 (*not satisfied at all*) through 10 (*very satisfied*).

#### Physical domain

At T2, sleep problems were assessed with the Basic Scale on Insomnia Complaints and Quality of Sleep (BaSIQS [40]). To asses adaptive physical functioning, emerging adults’ satisfaction concerning their general health was measured with one item on a ten-point scale with 1 (*not satisfied at all*) through 10 (*very satisfied*).

#### Self-concept domain

At T2, participants’ identity processes with reference to future plans were measured with the Dimensions of Identity Development Scale (DIDS [41]) divided into the adaptive outcomes commitment making, exploration in breadth, identification with commitment and exploration in depth, and the maladaptive outcome ruminative exploration. Emotional autonomy granted by parents was assessed with the Emotional Autonomy Scale (EAS [42]). As a good fit for a two-factor structure of the EAS has been found [43], we assessed emotional autonomy divided into two domains: separation as an adaptive outcome (reflecting a positive representation of the self and parents as separate individuals) and detachment as a maladaptive outcome (reflecting negative feelings toward parents, including alienation and distrust). The adaptive outcome self-efficacy was assessed with the Dutch translation of the General Self-Efficacy questionnaire [44].

#### Covariates

Background variables age of the child, child gender and family educational level were added as covariates in the main analyses. Family educational levels were 0.3% elementary school, 31.8% secondary school, 45.7% nonuniversity higher education, and 21.4% university or higher.

### Statistical analyses

To examine longitudinal associations between early internalizing and externalizing problems and emerging adulthood functioning, multiple bootstrapped regressions were performed in IBM Statistics SPSS 26.0. In the first step, background variables gender and age of the child and family educational level (as a continuous variable) were entered as covariates. In the second step, (mean-centered) main effects of internalizing problems and externalizing problems were entered. In the third step the interaction term of internalizing and externalizing problems was included. The confidence intervals and standardized errors were based on 1000 bootstrap samples. Reported estimates are the unstandardized beta coefficients and 95% bias corrected and accelerated confidence intervals in parentheses, associated standardized errors, standardized beta coefficients, and significance for each outcome based on a *p* value of < 0.05. To correct for multiple testing, we performed false discovery rate (FDR) analyses [45]. The adjusted *p* values for the associations that were significant (< 0.05) after FDR correction were reported. Effect sizes *r* were retrieved for the significant adjusted *p* values and interpreted with the following intervals for *r*: 0.10 and lower: no effect; 0.10–0.30: small effect; 0.30–0.50: intermediate effect; 0.50 and higher: strong effect [46].

## Results

Table 1 shows the means, standard deviations, and range of all variables of interest with mother and father data. Results of the multiple bootstrapped regressions for internalizing and externalizing problems, and their co-occurrence are described for each outcome variable in emerging adulthood, with mother data in Table 2 and father data in Table 3. Childhood internalizing and externalizing problems were moderately correlated (*r*_s_ mother reports = 0.58, *r*_s_ father reports = 0.60). Mother–father agreement was high for externalizing problems (*r*_s_ = 0.72) and moderate for internalizing problems (*r*_s_ = 63). Spearman correlations between the variables of interest are presented in Online Resource 1 and 2.

**Table 2:** Multiple bootstrapped regression analyses with mother reported early childhood internalizing problems, externalizing problems and their interactions and (mal)adaptive functioning in emerging adulthood

Outcome	Internalizing problems			Externalizing problems				Internalizing × externalizing problems		
	*B *(BCa CI)	*β*	SE* B*	*B *(BCa CI)	*β*	SE* B*	Adj*. p*^a^	*B *(BCa CI)	*β*	SE* B*
Psychological domain										
Internalizing dimension										
Anxious/depressed behaviour	0.19 (0.03, 0.35)*	0.13	0.08	0.10 (− 0.04, 0.23)	0.10	0.07		0.01 (− 0.01, 0.04)	0.16	0.01
Withdrawn behavior	0.04 (− 0.03, 0.11)	0.07	0.04	0.06 (0.01, 0.11)*	0.16	0.03		0.01 (0.002, 0.02)	0.23	0.01
Somatic complaints	0.05 (− 0.01, 0.12)	0.08	0.03	0.06 (0.01, 0.11)*	0.14	0.03		0.01 (− 0.003, 0.02)	0.21	0.01
Externalizing dimension										
Aggressive behavior	0.02 (− 0.07, 0.10)	0.03	0.05	0.10 (0.02, 0.18)**	0.18	0.04	0.037*	0.003 (− 0.01, 0.02)	0.06	0.01
Delinquency	− 0.04 (− 0.11, 0.03)	− 0.07	0.04	0.05 (− 0.004, 0.10)	0.12	0.03		− 0.001 (− 1.01, 0.01)	− 0.04	0.01
Intrusive behavior	− 0.01 (− 0.07, 0.04)	− 0.02	0.03	0.02 (− 0.03, 0.06)	0.05	0.02		− 0.01 (− 0.01, 0.003)	−0.17	0.004
Other problems										
Thought problems	0.02 (− 0.04, 0.07)	0.04	0.03	0.10 (0.06, 0.14)***	0.27	0.02	0.017*	0.01 (− 0.001, 0.02)	0.25	0.004
Attention problems	0.04 (− 0.09, 0.15)	0.04	0.06	0.11 (0.02, 0.19)*	0.15	0.04		0.01 (− 0.01, 0.02)	0.10	0.01
Personality pathology										
Negative affect	0.01 (− 0.01, 0.02)	0.07	0.01	0.01 (0.001, 0.02)*	0.14	0.001		0.001 (− 0.001, 0.002)	0.10	0.001
Detachment	0.001 (− 0.01, 0.01)	0.01	0.01	0.01 (0.01, 0.02)***	0.23	0.003		0.001 (− 0.001, 0.002)	0.12	0.001
Antagonism	−0.01 (− 0.02, 0.001)	− 0.09	0.01	0.01 (0.001, 0.02)*	0.13	0.004		− 0.001 (− 0.003, 0.00)	− 0.24	0.001
Disinhibition	− 0.01 (− 0.02, 0.01)	− 0.06	0.01	0.01 (0.001, 0.01)*	0.13	0.003		0.00 (− 0.001, 0.002)	0.04	0.001
Psychoticism	− 0.002 (− 0.01, 0.01)	− 0.02	0.01	0.01 (0.01, 0.02)*	0.21	0.01	0.015*	0.00 (− 0.001, 0.001)	− 0.04	0.001
Social domain										
Social problems	0.05 (− 0.02, 0.11)	0.08	0.03	0.09 (0.04, 0.14)**	0.21	0.03	0.042*	0.003 (− 0.01, 0.01)	0.08	0.004
Satisfaction friendships	− 0.01 (− 0.06, 0.03)	− 0.03	0.02	− 0.02 (− 0.05, 0.01)	− 0.09	0.02		0.001 (− 0.01, 0.01)	0.04	0.003
Satisfaction romantic relations	0.03 (− 0.03, 0.08)	0.05	0.03	− 0.06 (− 0.11, − 0.01)*	− 0.15	0.03		− 0.003 (− 0.01, 0.01)	− 0.09	0.01
Work domain										
Work satisfaction	0.001 (− 0.06, 0.07)	0.003	0.03	− 0.03 (− 0.07, 0.01)	− 0.13	0.02		0.002 (− 0.01, 0.01)	0.08	0.01
Physical domain										
Satisfaction general health	0.01 (− 0.03, 0.05)	0.03	0.02	− 0.03 (− 0.07, − 0.02)**	− 0.19	0.01	0.022*	− 0.001 (− 0.01, 0.01)	− 0.07	0.001
Sleep problems	− 0.01 (− 0.02, 0.01)	− 0.05	0.01	0.01 (− 0.002, 0.02)	0.10	0.01		− 0.001 (− 0.003, 0.001)	− 0.13	0.001
Self-concept domain										
Exploration in breadth	− 0.01 (− 0.03, 0.01)	− 0.06	0.01	− 0.003 (− 0.02, 0.01)	− 0.03	0.01		− 0.001 (− 0.003, 0.002)	− 0.09	0.001
Ruminative exploration	0.01 (− 0.02, 0.03)	0.03	0.01	0.02 (0.003, 0.03)*	0.14	0.01		0.001 (− 0.002, 0.004)	0.10	0.002
Identification with commitment	0.003 (− 0.02, 0.02)	0.02	0.01	− 0.01 (− 0.02, 0.01)	− 0.07	0.01		− 0.002 (− 0.01, 0.00)	− 0.24	0.001
Commitment making	0.003 (− 0.02, 0.03)	0.01	0.01	− 0.01 (− 0.03, 0.01)	− 0.09	0.01		− 0.001 (− 0.004, 0.002)	− 0.07	0.001
Exploration in depth	0.01 (− 0.01, 0.03)	0.08	0.01	− 0.01 (− 0.02, 0.002)	− 0.09	0.01		− 0.001 (− 0.004, 0.001)	− 0.13	0.001
Separation from parents	− 0.001 (− 0.01, 0.01)	− 0.01	0.01	− 0.01 (− 0.01, 0.001)	− 0.12	0.004		0.00 (− 0.001, 0.002)	0.01	0.001
Detachment from parents	− 0.001 (− 0.01, 0.01)	−0.01	0.01	− 0.01 (− 0.02, − 0.001)	− 0.13	0.01		0.00 (− 0.002, 0.002)	− 0.01	0.001
Self-efficacy	− 0.01 (− 0.02, 0.01)	− 0.06	0.01	− 0.002 (− 0.01, 0.01)	−0.04	0.04		− 0.001 (− 0.003, 0.00)	− 0.23	0.001

With control variables age and gender of the child, and family education

^a^Adjusted *p* values for false discovery rate (FDR)

*p < 0.05, **p < 0.01, ***p < 0.001

**Table 3:** Multiple bootstrapped regression analyses with father reported early childhood internalizing problems, externalizing problems and their interactions and (mal)adaptive functioning in emerging adulthood

Outcome	Internalizing problems			Externalizing problems				Internalizing x externalizing problems			
	*B (*BCa CI)	*β*	SE* B*	*B *(BCa CI)	*β*	SE* B*	Adj*. p*^a^	*B *(BCa CI)	*β*	SE* B*	Adj*. p*^a^
Psychological domain											
Internalizing dimension											
Anxious/depressed behaviour	0.10 (− 0.11, 0.29)	0.16	0.10	− 0.08 (− 0.05, 0.24)	0.08	0.07		− 0.01 (− 0.03, 0.01)	− 0.08	0.01	
Withdrawn behavior	0.02 (− 0.09, 0.11)	0.03	0.05	− 0.04 (− 0.02, 0.11)	0.10	0.03		0.00 (− 0.01, 0.01)	0.01	0.01	
Somatic complaints	0.03 (− 0.05, 0.12)	0.05	0.04	− 0.04 (− 0.02, 0.09)	0.09	0.03		− 0.01 (− 0.01, 0.01)	− 0.07	0.004	
Externalizing dimension											
Aggressive behavior	− 0.03 (− 0.13, 0.06)	− 0.04	0.05	− 0.07 (− 0.01, 0.14)	0.12	0.04		− 0.01 (− 0.02, − 0.004)	− 0.13	0.01	0.049*
Delinquency	− 0.01 (− 0.08, 0.08)	− 0.02	0.04	− 0.02 (− 0.03, 0.06)	0.05	0.02		0.001 (− 0.01, 0.01)	− 0.01	0.004	
Intrusive behavior	− 0.01 (− 0.07, 0.06)	− 0.03	0.03	− 0.02 (− 0.03, 0.08)	0.06	0.02		− 0.01 (− 0.01, 0.003)	− 0.11	0.004	
Other problems											
Thought problems	− 0.02 (− 0.09, 0.05)	− 0.03	0.03	− 0.06 (0.02, 0.11)**	0.16	0.02	0.029*	− 0.001 (− 0.01, 0.01)**	− 0.02	0.004	
Attention problems	− 0.02 (− 0.17, 0.12)	− 0.02	0.07	− 0.09 (− 0.02, 0.19)	0.12	0.05		0.00 (− 0.01, 0.02)	− 0.001	0.01	
Personality pathology											
Negative affect	0.002 (− 0.01, 0.01)	0.02	0.01	− 0.01 (− 0.001, 0.02)*	0.14	0.01		− 0.001 (− 0.002, 0.001)	− 0.05	0.001	
Detachment	− 0.001 (− 0.01, 0.01)	− 0.01	0.01	− 0.01 (0.004, 0.02)**	0.19	0.004	0.044*	− 0.001 (− 0.002, 0.001)	− 0.07	0.001	
Antagonism	− 0.01 (− 0.02, 0.00)	− 0.10	0.01	− 0.01 (− 0.003, 0.01)	0.08	0.004		− 0.001 (− 0.003, 0.00)	− 0.13	0.001	
Disinhibition	− 0.01 (− 0.02, 0.00)	− 0.10	0.01	− 0.01 (− 0.001, 0.01)	0.10	0.003		− 0.001 (− 0.001, 0.001)	− 0.004	0.001	
Psychoticism	− 0.01 (− 0.02, 0.00)	− 0.08	0.01	− 0.01 (0.004, 0.02)**	0.19	0.004	0.034*	− 0.001 (− 0.002, 0.001)	− 0.03	0.001	
Social domain											
Social problems	0.01 (− 0.07, 0.09)	0.02	0.04	− 0.06 (0.01, 0.12)*	0.15	0.03		0.03 (− 0.01, 0.01)	− 0.05	0.004	
Satisfaction friendships	0.01 (− 0.03, 0.06)	0.03	0.02	− 0.02 (− 0.07, 0.02)	− 0.11	0.02		0.02 (− 0.01, 0.01)	0.01	0.004	
Satisfaction romantic relations	− 0.01 (− 0.10. 0.07)	− 0.02	0.04	− 0.04 (− 0.09, 0.01)	− 0.09	0.03		0.03 (− 0.01, 0.01)	0.03	0.01	
Work domain											
Work satisfaction	− 0.04 (− 0.11, 0.07)	− 0.11	0.04	− 0.02 (− 0.08, 0.02)	− 0.11	0.03		0.03 (− 0.01, 0.01)	0.02	0.01	
Physical domain											
Satisfaction general health	0.03 (− 0.02, 0.07)	0.08	0.02	− 0.03 (− 0.06, 0.001)	− 0.13	0.02		0.02 (− 0.01, 0.01)	0.001	0.003	
Sleep problems	− 0.01 (− 0.03, 0.01)	− 0.08	0.01	0.01 (0.00, 0.02)*	0.14	0.01		0.01 (0.00, 0.00)	− 0.001	0.001	
Self-concept domain											
Exploration in breadth	− 0.01 (− 0.04, 0.01)	− 0.08	0.01	0.01 (− 0.01, 0.02)	0.07	0.01		0.00 (− 0.003, 0.002)	− 0.03	0.001	
Ruminative exploration	0.004 (− 0.03, 0.03)	0.13	0.01	0.02 (0.00, 0.04)	0.02	0.01		− 0.003 (− 0.01, 0.001)*	− 0.13	0.001	
Identification with commitment	0.01 (− 0.01, 0.04)	− 0. 06	0.01	− 0.01 (− 0.02, 0.01)	0.06	0.01		0.00 (− 0.003, 0.003)	0.01	0.002	
Commitment making	0.01 (− 0.02, 0.03)	− 0.08	0.01	− 0.01 (− 0.03, 0.01)	0.04	0.01		0.001 (− 0.002, 0.004)	0.06	0.001	
Exploration in depth	0.001 (− 0.02, 0.02)	0.001	0.01	0.001 (− 0.01, 0.01)	0.001	0.01		− 0.001 (− 0.003, 0.002)	− 0.05	0.001	
Separation from parents	− 0.004(− 0.02, 0.01)	− 0.01	0.01	− 0.001 (− 0.01, 0.01)	0.04	0.004		0.00 (− 0.001, 0.001)	− 0.03	0.001	
Detachment from parents	0.00 (− 0.01, 0.01)	− 0.03	0.01	− 0.002 (− 0.01, 0.01)	0.001	0.01		0.001 (0.00, 0.002)	0.08	0.001	
Self-efficacy	0.01 (− 0.02, 0.004)	− 0.01	0.01	− 0.001 (− 0.01, 0.01)	− 0.09	0.004		− 0.001 (− 0.002, 0.00)	− 0.12	0.001	

With control variables age and gender of the child, and family education

^a^Adjusted *p* values for false discovery rate (FDR)

*p < 0.05, **p < 0.01, ***p < 0.001

### Mother-reported early internalizing and externalizing problems and emerging adulthood functioning

*Early internalizing problems and emerging adulthood functioning.* Internalizing problems were not significantly related to any outcome in emerging adulthood.

*Early externalizing problems and emerging adulthood functioning. *Externalizing problems were associated with outcomes in the psychological, social, and physical domains of functioning in emerging adulthood. Regarding the psychological domain, externalizing problems were associated with aggressive behavior (*β* = 0.18, *p* < 0.05) with a small effect size (*r* = 0.23). Externalizing problems were related to more thought problems (*β* = 0.27, *p* < 0.05) with an intermediate effect size (*r* = 0.32). With regard to pathological personality dimensions, externalizing problems were related to psychoticism (*β* = 0.21, *p* < 0.05) with a small effect size of (*r* = 0.26). Moreover, for functioning within the social domain, early externalizing problems were significantly associated with more social problems (*β* = 0.21, *p* < 0.05) with a small effect size of *r* = 0.26. Within the physical domain, externalizing problems were negatively related to general health satisfaction (*β* = − 0.19, *p* < 0.05), with a small effect size (*r* = 0.24).

*Interaction of internalizing and externalizing problems and emerging adulthood functioning.* No significant interaction effects were found for internalizing and externalizing problems and emerging adulthood functioning.

### Father-reported early internalizing and externalizing problems and emerging adulthood functioning

*Early internalizing problems and emerging adulthood functioning.* Internalizing problems were not related to emerging adulthood functioning.

*Early externalizing problems and emerging adulthood functioning. *Externalizing problems were associated with outcomes in the psychological and physical domains of emerging adulthood functioning. In the psychological domain, externalizing problems were associated with thought problems (*β* = 0.16, *p* < 0.05) with a small effect size (*r* = 0.21). Significant relations were also found for pathological personality dimensions detachment (*β* = 0.19, *p* < 0.05) and psychoticism (*β* = 0.19, *p* < 0.05), both with small effect sizes (*r* = 0.24).

*Interaction of early internalizing and externalizing problems and emerging adulthood functioning.* A significant interaction effect was found for internalizing and externalizing problems and aggressive behavior in emerging adulthood (*β* = − 0.13, *p* < 0.01), with a small effect size (*r* = 0.18). The negative association between externalizing problems and aggressive behavior was strongest for lower scores on early internalizing problems and not significant for higher scores on early internalizing problems^1^.

## Discussion

The current study investigated whether additive and interactive effects of early childhood internalizing and externalizing problems were related to (mal)adaptive general functioning on a time span of 16 years. Major findings were that, after accounting for relevant covariates, early externalizing problems were associated with both maladaptive and adaptive emerging adulthood functioning outcomes on the psychological, social, and physical domains. With regard to the psychological domain, externalizing problems were related to more aggressive behavior from the externalizing dimension. This finding indicates long-term continuity from early childhood into emerging adulthood and the presence of a homotypic pattern (i.e., one type of problem predicts the same problem), similarly to earlier studies [1, 17, 18]. Early externalizing problems were not only related to the same type of problem domains, but also to outcomes on the social and physical domains. These findings emphasize the importance to extend the range of domains when examining long-term associations of internalizing and externalizing problems on maladaptive and adaptive functioning.

Some of the longitudinal associations for externalizing problems deserve more elaboration, as they were relatively strong considering the time span of 16 years and were significant in analyses of both mother-reported and father-reported early externalizing problems. Early externalizing problems were associated with pathological personality. There are two reasons that may explain the link to personality pathology: first, based on the scarring association [47] externalizing problems may maladaptively modify personality on the long term, in such a way that children with externalizing problems show more detached and more psychotic personalities in emerging adulthood. Possible explanatory factors, for example negative emotionality [48], for this scarring association with externalizing problems should be investigated further. Secondly, literature suggests that personality pathology and externalizing behavior share common behavioral and neurological disruptions [49], such as poor self-regulation. These long-term associations are important contributions of the current study, because personality pathology is associated with a poorer quality of life [30]. Furthermore, we found that early externalizing problems were related to more thought problems in emerging adulthood. Thought problems are characterized by strange thoughts and behaviors, hallucinations, self-harm, and suicide attempts. Thought problems are associated with many psychiatric disorders such as bipolar disorders [50] or obsessive–compulsive disorder [51]. Our results shed light on externalizing problems as early childhood precursors of personality pathology and thought problems in emerging adulthood.

Our multi-faceted approach of emerging adulthood functioning forms a strong asset of the current study. Additionally, the use of parent reports for childhood measures and emerging adults’ self-reported data allowed single-informant bias to be reduced [52]. Our findings indicated that early childhood externalizing problems are associated with similar developmental outcomes (homotypic pattern) in emerging adulthood, but provided also empirical evidence for a heterotypic pattern (i.e., longitudinal associations with other domains). However, we do caution against deterministic interpretations of our findings on the longitudinal associations of externalizing problems. Children can follow multiple paths to functioning outcomes in emerging adulthood. Individual or contextual risk or resilience factors, such as personality or parenting [35, 53], can strengthen or weaken these links to later functioning.

Our findings indicated that early internalizing problems were not related to any outcome in emerging adulthood. A first explanation points toward a concept of recovery with regard to internalizing problems. Earlier studies suggested internalizing problems have a higher tendency to recover by the time of emerging adulthood compared to externalizing problems [54]. A second explanation concerns the timing of the assessment of internalizing problems. According to earlier studies, the highest stability for externalizing problems was found from childhood and adolescence into emerging adulthood, whereas for internalizing problems stability is highest from adolescence onward [16, 55]. Thirdly, internalizing problems might have been underreported by parents compared to externalizing problems. Earlier studies reported higher visibility of externalizing problems in children as they involve conflicts with the environment, whereas internalizing problems involve conflicts within the child that are less visible to others [56]. Therefore, internalizing problems are less observed by parents. Thus, the fact that the parents reported internalizing problems of their child might (partly) account for the limited number of significant associations for internalizing problems. In our study, the assessment of internalizing and externalizing problems was based on the widely used instruments from the ASEBA system and the earliest age at which children report on their own internalizing and externalizing problems in the ASEBA system is the age of 11 years onward by the use of the YSR [38]. Of note, our replication of the mother-reported data with father-reported data on childhood problems adds to the robustness of our findings [54]. Two other reasons are possible for why internalizing problems might have been underreported. The first refers to internalizing and externalizing problems sharing some etiological factors, such as irritability. Irritablity is a transdiagnostic concept and refers to easy annoyance and touchiness [57]. According to the DSM-5, irritability is a symptom of disruptive mood dysregulation disorder and major depressive disorder in children, both internalizing disorders [57, 58]. Irritability is also a symptom of the externalizing disorder oppositional defiant disorder. Yet, irritability can be mistakenly attributed to externalizing problems in children, because the behavioral manifestations of irritability (temper outbursts) are more visible for other informants. The second issue concerns studies that found that symptoms for depression in adults differ from those in children [59]. As children show more somatic complaints, more irritability, and less signs of depression and anxiety compared to adults, children with depression are often less likely to meet the DSM-5 criteria for depression [59]. Hence, it is likely that some children that experience internalizing problems remain undetected or are mistakenly diagnosed with externalizing problems.

In addition to the additive effects of internalizing and externalizing problems, the current study also examined their interactive effects through a continuous approach. In contrast to the discrete approach, this approach allowed us to acknowledge the importance of cases that would fall just under the (borderline) clinical cutoff point for co-occurrence and would thus be undetected otherwise, but are of importance for our study of associations with functioning outcomes. The current study experienced difficulty of retrieving interactions of internalizing and externalizing problems, which appears to be the case more often for continuous variables in multiple regression analyses [60, 61]. The interaction term of (father reported) internalizing and externalizing problems was related to only one emerging adulthood outcome, i.e., aggressive behavior. Specifically, the negative association between externalizing problems in early childhood and aggressive behavior in emerging adulthood was strongest in the presence of lower internalizing problems in early childhood. We speculate that the longitudinal association between the interaction of internalizing and externalizing problems and aggressive behavior might depend on the level of internalizing problems in twofold: (1) as mentioned earlier, with regard to possible underreporting of internalizing problems and based on findings of previous studies [54], there is a possibility that some children labeled with externalizing problems would have been labeled as having internalizing problems later in life; (2) the pathoplastic theory suggests that some personality characteristics that are related to internalizing problems (i.e., decreased impulsivity) can explain how internalizing problems can exert positive influences on externalizing problems [47]. If internalizing problems were to be underreported, it may indicate that our results are in fact an underestimation of associations with (mal)adaptive functioning in emerging adulthood.

### Adaptive emerging adulthood functioning

Besides the often studied maladaptive functioning, the current study also addressed longitudinal relations between childhood internalizing and externalizing problems and developmental domains of adaptive functioning. Findings indicated that lower levels of (mother reported) externalizing problems in childhood were related to higher satisfaction concerning the quality of general health. General health is affected by functioning on other domains as well, such as psychological health and how one is functioning with regard to social relations [62]. In our study, externalizing problems were not only related to general health satisfaction, but also other maladaptive outcomes on different domains, such as psychological problems. Thus, it is most likely that emerging adults with more externalizing problems in their early childhood were less satisfied with their overall general health, because they also experienced various problems on other domains as well.

### Limitations and future directions

Several limitations of this study should be mentioned. Firstly, the sample consisted of relatively highly educated and only Caucasian participants. Previous research showed cultural differences in the prevalence of internalizing and externalizing problems [63, 64]. Possible cultural differences in the prevalence and consequences of early childhood internalizing and externalizing problems could not be disentangled. A second limitation concerns a methodological issue. The use of solely questionnaire measures may have increased the likelihood of method bias among our measures. Finally, we did not examine underlying mediating or moderating mechanisms or developmental pathways of the link between early internalizing and externalizing problems and emerging adulthood outcomes. In future studies, underlying mechanisms or developmental pathways of the link between early internalizing and externalizing problems and emerging adulthood outcomes might be explored based on a more person-centered strategy. Associations of stability and change with emerging adulthood functioning can be investigated for different profiles of internalizing and externalizing problems which are retrieved through Latent Profile Analysis and Latent Transition Analysis [65]. A person-centered approach may specifically provide more insight into the possibility for the tendency to recover from internalizing problems by the time of emerging adulthood and its implications for emerging adulthood functioning. Moreover, parenting quality, exposure to early adversities or trauma, and (child and parent) personality might play a role [8, 35, 53]. In future research, specifically the mediating role of parenting quality and the moderating role of early adversity and/or personality in the longitudinal associations could be examined.

## Conclusion

To our knowledge, the present study is the first to examine additive and interactive associations of early childhood internalizing and externalizing problems with both maladaptive and adaptive general functioning in emerging adulthood. The findings of this study contribute to our understanding of long-term associations of childhood internalizing and externalizing problems as developmental precursors of emerging adulthood functioning. Results emphasize the importance of prevention and intervention programs that are implemented at an early age and emphasize the value for developing personalized interventions*.* Future research should focus on underlying mediating and moderating mechanisms within the developmental pathways from early childhood onwards to further unravel the pathway into successful, healthy and happy emerging adulthood.

## Electronic supplementary material

Below is the link to the electronic supplementary material.

Supplementary file1 (DOCX 59 kb)

Supplementary file2 (DOCX 58 kb)

Supplementary file3 (DOCX 58 kb)

## Footnotes

1 Supplementary analyses were performed to explore moderation effects for child gender in the association between early childhood internalizing problems, externalizing problems, and their co-occurrence and (mal)adaptive functioning in emerging adulthood (see Online Resources 3–6). Multiple bootstrapped regression analyses with interaction terms for gender with internalizing and externalizing problems were performed with the mother reported and father reported data at T1. No significant moderation effects for child gender were found.
