# Supplementary material for: Early childhood internalizing problems, externalizing problems and their co-occurrence and (mal)adaptive functioning in emerging adulthood: a 16-year follow-up study
Source: Soc Psychiatry Psychiatr Epidemiol. 2020 Sep 22;56(2):193–206. doi: 10.1007/s00127-020-01959-w (PMC7870752; doi:10.1007/s00127-020-01959-w)
Supplement: Supplementary file 1 — Supplementary file1 (DOCX 59 kb) [file 127_2020_1959_MOESM1_ESM.docx]

**Early Childhood Internalizing Problems, Externalizing Problems and Their Co-Occurrence and (Mal)Adaptive Functioning in Emerging Adulthood:**

**A 16-Year Follow-Up Study**

*Journal of Social Psychiatry and Psychiatric Epidemiology*

İldeniz B. Arslan^*1^, Nicole Lucassen^1^, Pol A. C. van Lier^1 2^, Amaranta D. de Haan^1^, & Peter Prinzie^1^

^1^ Department of Psychology, Education and Child Studies, Erasmus University Rotterdam, Burgemeester Oudlaan 50, 3000 DR Rotterdam, the Netherlands.

^2^ Department of Clinical, Neuro and Developmental Psychology, VU University Amsterdam, De Boelelaan 1105, 1081 HV Amsterdam, the Netherlands.

**^*^**Corresponding author information: İldeniz B. Arslan, Burgemeester Oudlaan 50, 3000 DR Rotterdam, the Netherlands, (e-mail: arslan@essb.eur.nl), ORCID: 0000-0002-0967-578X.

Online Resource 1

|  | 1 | 2 | 3 | 4 | 5 | 6 | 7 | 8 | 9 | 10 | 11 | 12 |
| --- | --- | --- | --- | --- | --- | --- | --- | --- | --- | --- | --- | --- |
| 1. Early internalizing problems |  |  |  |  |  |  |  |  |  |  |  |  |
| 1. Early externalizing problems | .58^**^ |  |  |  |  |  |  |  |  |  |  |  |
| 1. Anxious/depressed behavior | .15^**^ | .10 |  |  |  |  |  |  |  |  |  |  |
| 1. Withdrawn behavior | .12^*^ | .16^**^ | .58^**^ |  |  |  |  |  |  |  |  |  |
| 1. Somatic complaints | .14^**^ | .14^**^ | .55^**^ | .33^**^ |  |  |  |  |  |  |  |  |
| 1. Aggressive behavior | .15^**^ | .18^**^ | .62^**^ | .49^**^ | .44^**^ |  |  |  |  |  |  |  |
| 1. Delinquency | .02 | .12^*^ | .24^**^ | .40^**^ | .27^**^ | .46^**^ |  |  |  |  |  |  |
| 1. Intrusive behavior | .04 | .08 | .08 | -.01 | .19^**^ | .41^**^ | .40^**^ |  |  |  |  |  |
| 1. Thought problems | .16^**^ | .26^**^ | .58^**^ | .54^**^ | .43^**^ | .57^**^ | .41^**^ | .27^**^ |  |  |  |  |
| 1. Attention problems | .12^*^ | .17^**^ | .43^**^ | .45^**^ | .36^**^ | .49^**^ | .51^**^ | .30^**^ | .49^**^ |  |  |  |
| 1. Negative affect | .12^*^ | .18^**^ | .71^**^ | .60^**^ | .45^**^ | .66^**^ | .43^**^ | .28^**^ | .62^**^ | .52^**^ |  |  |
| 1. Detachment | .09 | .24^**^ | .57^**^ | .69^**^ | .32^**^ | .52^**^ | .44^**^ | .15^**^ | .55^**^ | .47^**^ | .76^**^ |  |
| 1. Antagonism | .03 | .16^**^ | .12^*^ | .27^**^ | .15^**^ | .40^**^ | .46^**^ | .62^**^ | .37^**^ | .39^**^ | .50^**^ | .46^**^ |
| 1. Disinhibition | .02 | .14^**^ | .14^**^ | .29^**^ | .13^*^ | .34^**^ | .58^**^ | .38^**^ | .34^**^ | .67^**^ | .41^**^ | .39^**^ |
| 1. Psychoticism | .12^*^ | .26^**^ | .41^**^ | .52^**^ | .32^**^ | .50^**^ | .51^**^ | .28^**^ | .66^**^ | .51^**^ | .63^**^ | .66^**^ |
| 1. Social problems | .19^**^ | .21^**^ | .67^**^ | .60^**^ | .44^**^ | .60^**^ | .43^**^ | .25^**^ | .60^**^ | .53^**^ | .63^**^ | .54^**^ |
| 1. Satisfaction friendships | -.07 | -.10^*^ | -.29^**^ | -.38^**^ | -.08 | -.16^**^ | .01 | .09 | -.13^*^ | -.11^*^ | -.24^**^ | -.35^**^ |
| 1. Satisfaction romantic relations | -.02 | -.12^*^ | -.39^**^ | -.38^**^ | -.19^**^ | -.20^**^ | -.16^**^ | -.01 | -.26^**^ | -.20^**^ | -.31^**^ | -.41^**^ |
| 1. Work satisfaction | -.06 | -.15 | -.18^*^ | -.08 | -.08 | -.19^*^ | -.11 | -.11 | -.04 | -.16 | -.20^*^ | -.08 |
| 1. Satisfaction general health | -.07 | -.15^**^ | -.30^**^ | -.16^**^ | -.28^**^ | -.23^**^ | -.05 | -.03 | -.15^**^ | -.22^**^ | -.22^**^ | -.19^**^ |
| 1. Sleep problems | .02 | .07 | .23^**^ | .13^*^ | .37^**^ | .23^**^ | .07 | .06 | .17^**^ | .17^**^ | .21^**^ | .15^**^ |
| 1. Exploration in breadth | -.06 | -.08 | -.02 | -.08 | .00 | .00 | .01 | .01 | .05 | .03 | -.04 | -.06 |
| 1. Ruminative exploration | .08 | .14^**^ | .49^**^ | .35^**^ | .33^**^ | .33^**^ | .13^*^ | .07 | .37^**^ | .29^**^ | .47^**^ | .47^**^ |
| 1. Identification with commitment | .00 | -.02 | -.40^**^ | -.35^**^ | -.17^**^ | -.19^**^ | -.10 | .03 | -.24^**^ | -.25^**^ | -.32^**^ | -.37^**^ |
| 1. Commitment making | -.05 | -.10 | -.31^**^ | -.26^**^ | -.19^**^ | -.17^**^ | -.09 | -.03 | -.23^**^ | -.20^**^ | -.27^**^ | -.30^**^ |
| 1. Exploration in depth | .05 | -.03 | -.07 | -.26^**^ | -.01 | -.04 | -.12^*^ | .11^*^ | -.07 | -.10^*^ | -.09 | -.19^**^ |
| 1. Separation from parents | -.07 | -.13^*^ | -.19^**^ | -.27^**^ | -.13^*^ | -.26^**^ | -.25^**^ | -.05 | -.26^**^ | -.16^**^ | -.18^**^ | -.19^**^ |
| 1. Detachment from parents | -.08 | -.15^**^ | -.27^**^ | -.30^**^ | -.20^**^ | -.34^**^ | -.29^**^ | -.18^**^ | -.37^**^ | -.28^**^ | -.35^**^ | -.37^**^ |
| 1. Self-efficacy | -.06 | -.04 | -.41^**^ | -.20^**^ | -.17^**^ | -.25^**^ | -.04 | .05 | -.22^**^ | -.20^**^ | -.31^**^ | -.19^**^ |

*Correlations Between Variables of Interest, with Mother Data.*

Note. * *p* < .05, ** *p* < .01.

Online Resource 1 Continued

|  |  |  |  |  |  |  |  |  |  |  |  |  |  |  |  |  |
| --- | --- | --- | --- | --- | --- | --- | --- | --- | --- | --- | --- | --- | --- | --- | --- | --- |
| 1. Disinhibition | .51^**^ |  |  |  |  |  |  |  |  |  |  |  |  |  |  |  |
| 1. Psychoticism | .56^**^ | .50^**^ |  |  |  |  |  |  |  |  |  |  |  |  |  |  |
| 1. Social problems | .30^**^ | .34^**^ | .51^**^ |  |  |  |  |  |  |  |  |  |  |  |  |  |
| 1. Satisfaction friendships | -.02 | -.05 | -.12^*^ | -.20^**^ |  |  |  |  |  |  |  |  |  |  |  |  |
| 1. Satisfaction romantic relations | -.12^*^ | -.10 | -.22^**^ | -.27^**^ | .16^**^ |  |  |  |  |  |  |  |  |  |  |  |
| 1. Work satisfaction | -.15 | -.24^**^ | -.03 | -.07 | .10 | .20^*^ |  |  |  |  |  |  |  |  |  |  |
| 1. Satisfaction general health | .00 | -.11^*^ | -.12^*^ | -.26^**^ | .19^**^ | .20^**^ | .33^**^ |  |  |  |  |  |  |  |  |  |
| 1. Sleep problems | .01 | .06 | .09 | .17^**^ | -.05 | -.10 | -.22^*^ | -.26^**^ |  |  |  |  |  |  |  |  |
| 1. Exploration in breadth | -.05 | -.03 | .07 | -.04 | .02 | .05 | .00 | .07 | -.02 |  |  |  |  |  |  |  |
| 1. Ruminative exploration | .18^**^ | .17^**^ | .31^**^ | .34^**^ | -.16^**^ | -.33^**^ | -.32^**^ | -.14^**^ | .23^**^ | .00 |  |  |  |  |  |  |
| 1. Identification with commitment | -.04 | -.16^**^ | -.19^**^ | -.28^**^ | .19^**^ | .30^**^ | .13 | .18^**^ | -.12^*^ | .22^**^ | -.59^**^ |  |  |  |  |  |
| 1. Commitment making | -.11^*^ | -.15^**^ | -.20^**^ | -.22^**^ | .12^*^ | .32^**^ | .20^*^ | .17^**^ | -.21^**^ | .21^**^ | -.64^**^ | .69^**^ |  |  |  |  |
| 1. Exploration in depth | -.03 | -.15^**^ | -.10 | -.10 | .13^*^ | .12^*^ | -.03 | .04 | -.05 | .40^**^ | -.13^*^ | .43^**^ | .35^**^ |  |  |  |
| 1. Separation from parents | -.16^**^ | -.18^**^ | -.24^**^ | -.18^**^ | .05 | .14^**^ | .24^**^ | .07 | -.16^**^ | .00 | -.20^**^ | .17^**^ | .17^**^ | .09 |  |  |
| 1. Detachment from parents | -.32^**^ | -.28^**^ | -.46^**^ | -.35^**^ | .06 | .10^*^ | .16 | .17^**^ | -.16^**^ | -.03 | -.20^**^ | .14^**^ | .10^*^ | .01 | .40^**^ |  |
| 1. Self-efficacy | .14^**^ | -.04 | -.08 | -.30^**^ | .17^**^ | .07 | .02 | .23^**^ | -.11^*^ | .17^**^ | -.20^**^ | .30^**^ | .13^*^ | .17^**^ | -.01 | .06 |

Note. * *p* < .05, ** *p* < .01.

Online Resource 2

|  | 1 | 2 | 3 | 4 | 5 | 6 | 7 | 8 | 9 | 10 | 11 | 12 |
| --- | --- | --- | --- | --- | --- | --- | --- | --- | --- | --- | --- | --- |
| 1. Early internalizing problems |  |  |  |  |  |  |  |  |  |  |  |  |
| 1. Early externalizing problems | .60^**^ |  |  |  |  |  |  |  |  |  |  |  |
| 1. Anxious/depressed behavior | .07 | .12^*^ |  |  |  |  |  |  |  |  |  |  |
| 1. Withdrawn behavior | .03 | .12^*^ | .60^**^ |  |  |  |  |  |  |  |  |  |
| 1. Somatic complaints | .12^*^ | .17^**^ | .55^**^ | .33^**^ |  |  |  |  |  |  |  |  |
| 1. Aggressive behavior | .07 | .16^**^ | .63^**^ | .49^**^ | .46^**^ |  |  |  |  |  |  |  |
| 1. Delinquency | .00 | .07 | .24^**^ | .40^**^ | .25^**^ | .45^**^ |  |  |  |  |  |  |
| 1. Intrusive behavior | .03 | .09 | .07 | -.02 | .18^**^ | .40^**^ | .38^**^ |  |  |  |  |  |
| 1. Thought problems | .08 | .17^**^ | .57^**^ | .54^**^ | .41^**^ | .57^**^ | .40^**^ | .26^**^ |  |  |  |  |
| 1. Attention problems | .04 | .12^*^ | .43^**^ | .47^**^ | .38^**^ | .50^**^ | .52^**^ | .29^**^ | .50^**^ |  |  |  |
| 1. Negative affect | .08 | .18^**^ | .71^**^ | .61^**^ | .44^**^ | .67^**^ | .43^**^ | .27^**^ | .62^**^ | .52^**^ |  |  |
| 1. Detachment | .06 | .21^**^ | .57^**^ | .70^**^ | .31^**^ | .52^**^ | .44^**^ | .14^*^ | .54^**^ | .47^**^ | .76^**^ |  |
| 1. Antagonism | -.02 | .10 | .10 | .26^**^ | .13^*^ | .39^**^ | .44^**^ | .62^**^ | .36^**^ | .39^**^ | .48^**^ | .44^**^ |
| 1. Disinhibition | -.03 | .08 | .14^**^ | .31^**^ | .14^**^ | .35^**^ | .58^**^ | .36^**^ | .35^**^ | .67^**^ | .41^**^ | .40^**^ |
| 1. Psychoticism | .05 | .19^**^ | .41^**^ | .53^**^ | .30^**^ | .49^**^ | .51^**^ | .27^**^ | .65^**^ | .52^**^ | .63^**^ | .66^**^ |
| 1. Social problems | .08 | .17^**^ | .67^**^ | .61^**^ | .43^**^ | .59^**^ | .42^**^ | .24^**^ | .59^**^ | .54^**^ | .63^**^ | .54^**^ |
| 1. Satisfaction friendships | .00 | -.04 | -.28^**^ | -.39^**^ | -.08 | -.16^**^ | .02 | .09 | -.13^*^ | -.11^*^ | -.25^**^ | -.37^**^ |
| 1. Satisfaction romantic relations | -.05 | -.12^*^ | -.40^**^ | -.39^**^ | -.17^**^ | -.22^**^ | -.17^**^ | -.01 | -.27^**^ | -.24^**^ | -.33^**^ | -.43^**^ |
| 1. Work satisfaction | -.08 | -.16 | -.18^*^ | -.09 | -.07 | -.21^*^ | -.11 | -.12 | -.05 | -.19^*^ | -.20^*^ | -.09 |
| 1. Satisfaction general health | .01 | -.11^*^ | -.31^**^ | -.18^**^ | -.29^**^ | -.25^**^ | -.04 | -.04 | -.17^**^ | -.23^**^ | -.24^**^ | -.21^**^ |
| 1. Sleep problems | .02 | .12^*^ | .26^**^ | .14^**^ | .38^**^ | .25^**^ | .05 | .04 | .20^**^ | .18^**^ | .23^**^ | .17^**^ |
| 1. Exploration in breadth | -.02 | .00 | -.05 | -.09 | .00 | -.01 | -.01 | .01 | .03 | .02 | -.05 | -.07 |
| 1. Ruminative exploration | .09 | .16^**^ | .49^**^ | .35^**^ | .32^**^ | .33^**^ | .13^*^ | .06 | .37^**^ | .30^**^ | .47^**^ | .46^**^ |
| 1. Identification with commitment | .01 | -.03 | -.41^**^ | -.37^**^ | -.19^**^ | -.20^**^ | -.12^*^ | .03 | -.25^**^ | -.25^**^ | -.33^**^ | -.38^**^ |
| 1. Commitment making | -.02 | -.10 | -.31^**^ | -.29^**^ | -.19^**^ | -.18^**^ | -.11^*^ | -.03 | -.24^**^ | -.20^**^ | -.28^**^ | -.33^**^ |
| 1. Exploration in depth | .00 | -.02 | -.09 | -.27^**^ | -.04 | -.05 | -.13^*^ | .12^*^ | -.08 | -.11^*^ | -.10 | -.21^**^ |
| 1. Separation from parents | -.01 | -.05 | -.19^**^ | -.28^**^ | -.13^*^ | -.25^**^ | -.24^**^ | -.03 | -.28^**^ | -.16^**^ | -.18^**^ | -.18^**^ |
| 1. Detachment from parents | -.03 | -.08 | -.28^**^ | -.34^**^ | -.22^**^ | -.35^**^ | -.31^**^ | -.18^**^ | -.41^**^ | -.28^**^ | -.35^**^ | -.38^**^ |
| 1. Self-efficacy | -.05 | -.03 | -.40^**^ | -.21^**^ | -.18^**^ | -.23^**^ | -.02 | .07 | -.21^**^ | -.19^**^ | -.31^**^ | -.19^**^ |

*Correlations Between Variables of Interest, with Father Data.*

Note. * *p* < .05, ** *p* < .01.

Online Resource 2 Continued

|  | 13 | 14 | 15 | 16 | 17 | 18 | 19 | 20 | 21 | 22 | 23 | 24 | 25 | 26 | 27 | 28 |
| --- | --- | --- | --- | --- | --- | --- | --- | --- | --- | --- | --- | --- | --- | --- | --- | --- |
| 14 Disinhibition | .51^**^ |  |  |  |  |  |  |  |  |  |  |  |  |  |  |  |
| 15 Psychoticism | .55^**^ | .53^**^ |  |  |  |  |  |  |  |  |  |  |  |  |  |  |
| 16 Social problems | .29^**^ | .35^**^ | .51^**^ |  |  |  |  |  |  |  |  |  |  |  |  |  |
| 17 Satisfaction friendships | -.02 | -.05 | -.13^*^ | -.19^**^ |  |  |  |  |  |  |  |  |  |  |  |  |
| 18 Satisfaction romantic relations | -.12^*^ | -.13^*^ | -.23^**^ | -.28^**^ | .17^**^ |  |  |  |  |  |  |  |  |  |  |  |
| 19 Work satisfaction | -.17 | -.25^**^ | -.06 | -.07 | .09 | .17 |  |  |  |  |  |  |  |  |  |  |
| 20 Satisfaction general health | -.02 | -.11^*^ | -.14^*^ | -.27^**^ | .19^**^ | .20^**^ | .31^**^ |  |  |  |  |  |  |  |  |  |
| 21 Sleep problems | -.01 | .06 | .11^*^ | .18^**^ | -.06 | -.09 | -.18^*^ | -.25^**^ |  |  |  |  |  |  |  |  |
| 22 Exploration in breadth | -.03 | -.04 | .05 | -.06 | .05 | .04 | -.01 | .09 | -.02 |  |  |  |  |  |  |  |
| 23 Ruminative exploration | .17^**^ | .18^**^ | .30^**^ | .34^**^ | -.17^**^ | -.33^**^ | -.34^**^ | -.15^**^ | .23^**^ | -.01 |  |  |  |  |  |  |
| 24 Identification with commitment | -.04 | -.16^**^ | -.20^**^ | -.29^**^ | .21^**^ | .33^**^ | .16 | .19^**^ | -.15^**^ | .25^**^ | -.60^**^ |  |  |  |  |  |
| 25 Commitment making | -.11^*^ | -.16^**^ | -.22^**^ | -.23^**^ | .14^**^ | .32^**^ | .19^*^ | .16^**^ | -.21^**^ | .22^**^ | -.65^**^ | .70^**^ |  |  |  |  |
| 26 Exploration in depth | -.02 | -.14^**^ | -.11^*^ | -.12^*^ | .15^**^ | .16^**^ | -.02 | .05 | -.07 | .42^**^ | -.15^**^ | .44^**^ | .38^**^ |  |  |  |
| 27 Separation from parents | -.13^*^ | -.18^**^ | -.25^**^ | -.18^**^ | .06 | .13^*^ | .25^**^ | .08 | -.15^**^ | -.01 | -.20^**^ | .19^**^ | .18^**^ | .10 |  |  |
| 28 Detachment from parents | -.31^**^ | -.29^**^ | -.49^**^ | -.37^**^ | .07 | .12^*^ | .16 | .17^**^ | -.17^**^ | -.04 | -.21^**^ | .15^**^ | .09 | .01 | .40^**^ |  |
| 29 Self-efficacy | .17^**^ | -.02 | -.08 | -.29^**^ | .15^**^ | .10 | .03 | .25^**^ | -.13^*^ | .20^**^ | -.20^**^ | .31^**^ | .14^**^ | .18^**^ | -.02 | .06 |

Note. * *p* < .05, ** *p* < .01.
