# Supplementary material for: Early childhood internalizing problems, externalizing problems and their co-occurrence and (mal)adaptive functioning in emerging adulthood: a 16-year follow-up study
Source: Soc Psychiatry Psychiatr Epidemiol. 2020 Sep 22;56(2):193–206. doi: 10.1007/s00127-020-01959-w (PMC7870752; doi:10.1007/s00127-020-01959-w)
Supplement: Supplementary file 2 — Supplementary file2 (DOCX 58 kb) [file 127_2020_1959_MOESM2_ESM.docx]

**Early Childhood Internalizing Problems, Externalizing Problems and Their Co-Occurrence and (Mal)Adaptive Functioning in Emerging Adulthood:**

**A 16-Year Follow-Up Study**

*Journal of Social Psychiatry and Psychiatric Epidemiology*

İldeniz B. Arslan^*1^, Nicole Lucassen^1^, Pol A. C. van Lier^1 2^, Amaranta D. de Haan^1^, & Peter Prinzie^1^

^1^ Department of Psychology, Education and Child Studies, Erasmus University Rotterdam, Burgemeester Oudlaan 50, 3000 DR Rotterdam, the Netherlands.

^2^ Department of Clinical, Neuro and Developmental Psychology, VU University Amsterdam, De Boelelaan 1105, 1081 HV Amsterdam, the Netherlands.

**^*^**Corresponding author information: İldeniz B. Arslan, Burgemeester Oudlaan 50, 3000 DR Rotterdam, the Netherlands, (e-mail: arslan@essb.eur.nl), ORCID: 0000-0002-0967-578X.

Online Resource 3

*Multiple Bootstrapped Regression Analyses^a^ with Mother Reported Early Childhood Internalizing Problems, Externalizing Problems*

*and Their Interactions and (Mal)Adaptive Functioning in Emerging Adulthood, For Girls.*

|  |  | Internalizing problems | | |  | Externalizing problems | | |  | Internalizing x externalizing problems | | |  |
| --- | --- | --- | --- | --- | --- | --- | --- | --- | --- | --- | --- | --- | --- |
| ***Outcome*** |  | *B(BCa CI)* | β | *SE* *B* |  | *B(BCa CI)* | β | *SE* *B* |  | *B(BCa CI)* | β | *SE* *B* |  |
| **Psychological domain** |  |  |  |  |  |  |  |  |  |  |  |  |  |
| *Internalizing dimension* |  |  |  |  |  |  |  |  |  |  |  |  |  |
| Anxious/depressed behavior |  | .16(-.10, .36) | .12 | .11 |  | .05(-.16, .27) | .04 | .11 |  | .01(-.02, .05) | .07 | .02 |  |
| Withdrawn behavior |  | .04(-.06, .13) | .08 | .05 |  | .03(-.03, .10) | .08 | .04 |  | .01(-.01, .02) | .09 | .01 |  |
| Somatic complaints |  | .07(-.02, .15) | .10 | .05 |  | .04(-.05, .12) | .07 | .05 |  | .004(-.01, .02) | .05 | .01 |  |
| *Externalizing dimension* |  |  |  |  |  |  |  |  |  |  |  |  |  |
| Aggressive behavior |  | -.01(-.15, .11) | -.01 | .07 |  | .11(-.01, .24) | .16 | .07 |  | .002(-.02, .02) | .02 | .01 |  |
| Delinquency |  | -.06(-.17, .02) | -.11 | .05 |  | .05(-.03, .15) | .12 | .05 |  | .003(-.01, .02) | .03 | .01 |  |
| Intrusive behavior |  | -.04(-.12, .01) | -.10 | .04 |  | .07(-.01, .16) | .18 | .04 |  | -.003(-.02, .01) | -.05 | .01 |  |
| *Other problems* |  |  |  |  |  |  |  |  |  |  |  |  |  |
| Thought problems |  | -.04(-.13, .04) | -.07 | .04 |  | .10(.02, .16)** | .24 | .03 |  | .01(-.01, .02) | .12 | .01 |  |
| Attention problems |  | -.02(-.15, .11) | -.02 | .07 |  | .12(-.02, .24) | .14 | .07 |  | .01(-.02, .03) | .05 | .01 |  |
| *Personality pathology* |  |  |  |  |  |  |  |  |  |  |  |  |  |
| Negative affect |  | .01(-.01, .02) | .05 | .01 |  | .01(-.01, .02) | .11 | .01 |  | .001(-.001, .003) | .08 | .001 |  |
| Detachment |  | -.004(-.01, .01) | -.05 | .01 |  | .01(.002, .02)* | .19 | .01 |  | .00(-.001, .002) | .04 | .001 |  |
| Antagonism |  | -.01(-.02, .01) | -.08 | .01 |  | .01(-.003, .02) | .14 | .01 |  | -.001(-.003, .001) | -.09 | .001 |  |
| Disinhibition |  | -.01(-.02, .01) | -.12 | .01 |  | .01(-.001, .02) | .15 | .01 |  | .001(-.001, .002) | .06 | .001 |  |
| Psychoticism |  | -.001(-.02, .01) | -.01 | .01 |  | .01(-.002, .02) | .15 | .01 |  | .00(-.002, .002) | .003 | .001 |  |
| **Social domain** |  |  |  |  |  |  |  |  |  |  |  |  |  |
| Social problems |  | .02(-.08, .10) | .04 | .05 |  | .09(.01, .19)* | .19 | .04 |  | .00(-.01, .01) | -.01 | .01 |  |
| Satisfaction friendships |  | .00(-.04, .05) | .01 | .03 |  | -.02(-.07, .02) | -.10 | .02 |  | -.001(-.01, .01) | -.03 | .004 |  |
| Satisfaction romantic relations |  | .04(-.05, .11) | .06 | .04 |  | -.05(-.12, .03) | -.10 | .04 |  | -.003(-.02, .01) | -.03 | .01 |  |
| **Work domain** |  |  |  |  |  |  |  |  |  |  |  |  |  |
| Work satisfaction |  | .04(-.07, .17) | .13 | .06 |  | -.06(-.16, .03) | -.21 | .05 |  | .01(-.01, .02) | .10 | .01 |  |
| **Physical domain** |  |  |  |  |  |  |  |  |  |  |  |  |  |
| Satisfaction general health |  | -.01(-.06, .05) | -.02 | .03 |  | -.03(-.09, .03) | -.12 | .03 |  | .00(-.01, .01) | -.003 | .01 |  |
| Sleep problems |  | -.01(-.02, .01) | -.05 | .01 |  | .00(-.01, .02) | .02 | .01 |  | -.002(.00, .001) | -.08 | .01 |  |
| **Self-concept domain** |  |  |  |  |  |  |  |  |  |  |  |  |  |
| Exploration in breadth |  | -.01(-.04, .01) | -.08 | .01 |  | -.01(-.03, .01) | -.07 | .01 |  | -.001(.01, .002) | -.06 | .002 |  |
| Ruminative exploration |  | -.01(-.04, .03) | -.03 | .02 |  | .02(-.01, .04) | .12 | .01 |  | .001(.01, .01) | .02 | .002 |  |
| Identification with commitment |  | .02(-.01, .05) | .12 | .01 |  | -.01(-.03, .01) | -.07 | .01 |  | -.003(-.01, .001) | -.15 | .003 |  |
| Commitment making |  | .01(-.02, .04) | .06 | .02 |  | -.01(-.04, .02) | -.07 | .01 |  | .00(.004, .01) | -.004 | .002 |  |
| Exploration in depth |  | .01(-.02, .03) | .06 | .01 |  | -.02(-.04, .01) | -.14 | .01 |  | -.003(-.01, .001) | -.15 | .002 |  |
| Separation from parents |  | -.003(-.02, .01) | -.04 | .01 |  | -.004(-.02, .01) | -.05 | .01 |  | .001(.002, .003) | .05 | .002 |  |
| Detachment from parents |  | .003(-.01, .02) | .03 | .01 |  | -.01(-.02, .01) | -.08 | .01 |  | .001(-.001, .001) | .05 | .001 |  |
| Self-efficacy |  | -.003(-.02, .02) | -.03 | .01 |  | -.003(-.02, .01) | -.04 | .01 |  | -.003(-.004, .001) | -.10 | .001 |  |

Note. *p < .05. ***p* < .01. No significant adjusted p-values for FDR were found, reported are significance values without FDR correction. ^a^ With control variables age of the child and family education.

Online Resource 4

*Multiple Bootstrapped Regression Analyses^a^ with Mother Reported Early Childhood Internalizing Problems, Externalizing Problems and Their Interactions and (Mal)Adaptive Functioning in Emerging Adulthood, For Boys.*

|  |  | Internalizing problems | | |  | Externalizing problems | | | | | | |  | | | Internalizing x externalizing problems | | | | | | |  | | |
| --- | --- | --- | --- | --- | --- | --- | --- | --- | --- | --- | --- | --- | --- | --- | --- | --- | --- | --- | --- | --- | --- | --- | --- | --- | --- |
| ***Outcome*** |  | *B(BCa CI)* | β | *SE* *B* |  | *B(BCa CI)* | β | *SE* *B* | | *Adj. p* ^b^ | |  | | | *B(BCa CI)* | | | β | | *SE* *B* | |  | | |  |
| **Psychological domain** |  |  |  |  |  |  |  |  |  | |  | | |  | | |  | |  | |  | | |  |  |
| *Internalizing dimension* |  |  |  |  |  |  |  |  |  | |  | | |  | | |  | |  | |  | | |  |  |
| Anxious/depressed behavior |  | .30(.00, .61)* | .19 | .14 |  | .11(-.05, .28) | .12 | .08 |  | |  | | | .01(-.03, .04) | | | .04 | | .02 | |  | | |  |  |
| Withdrawn behavior |  | .07(-.05, .18) | .10 | .06 |  | .08(.002, .16)* | .19 | .04 |  | |  | | | .01(-.01, .02) | | | .09 | | .01 | |  | | |  |  |
| Somatic complaints |  | .06(-.02, .16) | .10 | .05 |  | .07(.02, .13)** | .22 | .03 |  | |  | | | .01(-.004, .02) | | | .13 | | .01 | |  | | |  |  |
| *Externalizing dimension* |  |  |  |  |  |  |  |  |  | |  | | |  | | |  | |  | |  | | |  |  |
| Aggressive behavior |  | .07(-.04, .20) | .10 | .06 |  | .08(-.002, .16) | .19 | .04 |  | |  | | | .003(-.01, .02) | | | .03 | | .01 | |  | | |  |  |
| Delinquency |  | -.01(-.11, .13) | -.01 | .06 |  | .04(-.02, .10) | .12 | .03 |  | |  | | | -.01(-.02, .01) | | | -.07 | | .01 | |  | | |  |  |
| Intrusive behavior |  | .02(-.07, .11) | .04 | .05 |  | -.03(-.10, .03) | -.09 | .03 |  | |  | | | -.01(-.02, .002) | | | -.10 | | .01 | |  | | |  |  |
| *Other problems* |  |  |  |  |  |  |  |  |  | |  | | |  | | |  | |  | |  | | |  |  |
| Thought problems |  | .12(.03, .23)** | .21 | .05 |  | .09(.04, .14)*** | .26 | .03 | **.42*** | |  | | | .01(-.01, .02) | | | .06 | | .01 | |  | | |  |  |
| Attention problems |  | .12(-.08, .32) | .11 | .11 |  | .10(-.03, .22) | .14 | .06 |  | |  | | | .004(-.02, .03) | | | .03 | | .01 | |  | | |  |  |
| *Personality pathology* |  |  |  |  |  |  |  |  |  | |  | | |  | | |  | |  | |  | | |  |  |
| Negative affect |  | .01(-.01, .03) | .11 | .01 |  | .01(-.001, .02) | .15 | .01 |  | |  | | | .00(-.002, .002) | | | -.01 | | .001 | |  | | |  |  |
| Detachment |  | .01(-.01, .02) | .08 | .01 |  | .01(.01, .02)*** | .25 | .01 | **.44*** | |  | | | .001(-.001, .002) | | | .06 | | .001 | |  | | |  |  |
| Antagonism |  | -.01(-.03, .01) | -.10 | .01 |  | .01(-.01, .02) | .12 | .01 |  | |  | | | -.002(-.004, .00) | | | -.11 | | .001 | |  | | |  |  |
| Disinhibition |  | .00(-.01, .01) | .00 | .01 |  | .01(-.004, .01) | .10 | .004 |  | |  | | | .00(-.003, .002) | | | -.03 | | .001 | |  | | |  |  |
| Psychoticism |  | .00(-.01, .02) | .002 | .01 |  | .01(.01, .02)*** | .25 | .004 |  | |  | | | -.001(-.002, .001) | | | -.04 | | .001 | |  | | |  |  |
| **Social domain** |  |  |  |  |  |  |  |  |  | |  | | |  | | |  | |  | |  | | |  |  |
| Social problems |  | .09(-.01, .19) | .15 | .05 |  | .07(.01, .14)* | .20 | .03 |  | |  | | | .004(-.01, .02) | | | .05 | | .01 | |  | | |  |  |
| Satisfaction friendships |  | -.03(-.11, .04) | -.08 | .03 |  | -.02(-.05, .02) | -.08 | .02 |  | |  | | | .002(-.01, .01) | | | .05 | | .01 | |  | | |  |  |
| Satisfaction romantic relations |  | .00(-.10, .12) | .004 | .05 |  | -.08(-.13, -.01)* | -.20 | .03 |  | |  | | | -.003(-.02, .01) | | | -.03 | | .01 | |  | | |  |  |
| **Work domain** |  |  |  |  |  |  |  |  |  | |  | | |  | | |  | |  | |  | | |  |  |
| Work satisfaction |  | -.03(-.12, .06) | -.11 | .05 |  | -.01(-.07, .03) | -.07 | .03 |  | |  | | | -.001(-.02, .01) | | | -.03 | | .01 | |  | | |  |  |
| **Physical domain** |  |  |  |  |  |  |  |  |  | |  | | |  | | |  | |  | |  | | |  |  |
| Satisfaction general health |  | .03(-.02, .07) | .09 | .03 |  | -.05(-.08, -.02)** | -.27 | .01 | **.32*** | |  | | | -.002(-.01, .003) | | | -.06 | | .004 | |  | | |  |  |
| Sleep problems |  | .00(-.03, .02) | -.03 | .01 |  | .01(.002, .03)* | .19 | .01 |  | |  | | | .00(-.003, .002) | | | -.02 | | .001 | |  | | |  |  |
| **Self-concept domain** |  |  |  |  |  |  |  |  |  | |  | | |  | | |  | |  | |  | | |  |  |
| Exploration in breadth |  | .004(-.03, .04) | .02 | .02 |  | -.002(-.02, .01) | -.03 | .01 |  | |  | | | -.001(-.004, .002) | | | -.03 | | .002 | |  | | |  |  |
| Ruminative exploration |  | .02(-.01, .06) | .12 | .02 |  | .02(-.001, .04)* | .17 | .01 |  | |  | | | .001(-.004, .01) | | | .04 | | .002 | |  | | |  |  |
| Identification with commitment |  | -.02(-.05, .02) | -.10 | .01 |  | -.01(-.03, .01) | -.09 | .01 |  | |  | | | -.001(-.01, .003) | | | -.04 | | .002 | |  | | |  |  |
| Commitment making |  | -.01(-.05, .02) | -.06 | .02 |  | -.02(-.04, .01) | -.13 | .01 |  | |  | | | -.001(-.01, .004) | | | -.04 | | .002 | |  | | |  |  |
| Exploration in depth |  | .02(-.01, .06) | .13 | .02 |  | -.01(-.02, .01) | -.07 | .01 |  | |  | | | .001(-.002, .003) | | | .03 | | .001 | |  | | |  |  |
| Separation from parents |  | .003(-.01, .02) | .03 | .01 |  | -.01(-.02, -.001)* | -.20 | .004 |  | |  | | | .00(-.002, .002) | | | -.05 | | .001 | |  | | |  |  |
| Detachment from parents |  | -.004(-.02, .01) | -.03 | .01 |  | -.01(-.03, -.003)* | -.23 | .01 |  | |  | | | -.001(-.01, .002) | | | -.09 | | .002 | |  | | |  |  |
| Self-efficacy |  | -.01(-.03, .002)** | -.15 | .01 |  | .00(-.01, .01) | .01 | .01 |  | |  | | | .00(-.002, .001) | | | -.05 | | .001 | |  | | |  |  |

Note. *p < .05. ** *p* < .01. *** *p* < .001. ^a^ With control variables age of the child and family education. ^b^ Adjusted p-values for False Discovery Rate (FDR).
