# Supplementary material for: Early childhood internalizing problems, externalizing problems and their co-occurrence and (mal)adaptive functioning in emerging adulthood: a 16-year follow-up study
Source: Soc Psychiatry Psychiatr Epidemiol. 2020 Sep 22;56(2):193–206. doi: 10.1007/s00127-020-01959-w (PMC7870752; doi:10.1007/s00127-020-01959-w)
Supplement: Supplementary file 3 — Supplementary file3 (DOCX 58 kb) [file 127_2020_1959_MOESM3_ESM.docx]

**Early Childhood Internalizing Problems, Externalizing Problems and Their Co-Occurrence and (Mal)Adaptive Functioning in Emerging Adulthood:**

**A 16-Year Follow-Up Study**

*Journal of Social Psychiatry and Psychiatric Epidemiology*

İldeniz B. Arslan^*1^, Nicole Lucassen^1^, Pol A. C. van Lier^1 2^, Amaranta D. de Haan^1^, & Peter Prinzie^1^

^1^ Department of Psychology, Education and Child Studies, Erasmus University Rotterdam, Burgemeester Oudlaan 50, 3000 DR Rotterdam, the Netherlands.

^2^ Department of Clinical, Neuro and Developmental Psychology, VU University Amsterdam, De Boelelaan 1105, 1081 HV Amsterdam, the Netherlands.

**^*^**Corresponding author information: İldeniz B. Arslan, Burgemeester Oudlaan 50, 3000 DR Rotterdam, the Netherlands, (e-mail: arslan@essb.eur.nl), ORCID: 0000-0002-0967-578X.

Online Resource 5

*Multiple Bootstrapped Regression Analyses^a^ with Father Reported Early Childhood Internalizing Problems, Externalizing Problems and*

*Their Interactions and (Mal)Adaptive Functioning in Emerging Adulthood, For Girls.*

|  |  | Internalizing problems | | | |  | | Externalizing problems | | | |  | | Internalizing x externalizing problems | | | |  | |
| --- | --- | --- | --- | --- | --- | --- | --- | --- | --- | --- | --- | --- | --- | --- | --- | --- | --- | --- | --- |
| ***Outcome*** |  | *B(BCa CI)* | β | *SE* *B* |  | | *B(BCa CI)* | | β | *SE* *B* |  | | *B(BCa CI)* | | β | *SE* *B* |  | |  |
| **Psychological domain** |  |  |  |  |  | |  | |  |  |  | |  | |  |  |  | |  |
| *Internalizing dimension* |  |  |  |  |  | |  | |  |  |  | |  | |  |  |  | |  |
| Anxious/depressed behavior |  | .08(-.23, .31) | .05 | .14 |  | | .07(-.13, .31) | | .06 | .11 |  | | -.01(-.03, .06) | | -.03 | .02 |  | |  |
| Withdrawn behavior |  | -.01(-.15, .11) | -.02 | .07 |  | | .02(-.06, .11) | | .06 | .04 |  | | .00(-.01, .02) | | -.01 | .01 |  | |  |
| Somatic complaints |  | .02(-.10, .13) | .02 | .06 |  | | .04(-.05, .14) | | .08 | .05 |  | | -.001(-.01, .02) | | -.02 | .01 |  | |  |
| *Externalizing dimension* |  |  |  |  |  | |  | |  |  |  | |  | |  |  |  | |  |
| Aggressive behavior |  | -.13(-.28, .01) | -.14 | .07 |  | | .09(-.03, .23) | | .13 | .07 |  | | -.01(-.02, .01) | | -.05 | .01 |  | |  |
| Delinquency |  | -.04(-.13, .04) | -.07 | .04 |  | | -.004(-.07, .07) | | -.01 | .03 |  | | .00(-.01, .02) | | .04 | .01 |  | |  |
| Intrusive behavior |  | -.06(-.13, .04) | -.12 | .04 |  | | .04(-.02, .11) | | .12 | .04 |  | | -.01(-.02, .001)* | | -.16 | .01 |  | |  |
| *Other problems* |  |  |  |  |  | |  | |  |  |  | |  | |  |  |  | |  |
| Thought problems |  | -.06(-.14, .02) | -.11 | .04 |  | | .06(-.003, .12) | | .15 | .03 |  | | .00(-.01, .02) | | .01 | .01 |  | |  |
| Attention problems |  | -.13(-.30, .04) | -.12 | .08 |  | | .11(.002, .23) | | .15 | .06 |  | | .00(-.02, .02) | | .01 | .01 |  | |  |
| *Personality pathology* |  |  |  |  |  | |  | |  |  |  | |  | |  |  |  | |  |
| Negative affect |  | -.01(-.02, .01) | -.05 | .01 |  | | .01(.00, .03) | | .17 | .01 |  | | .00(-.002, .004) | | .02 | .001 |  | |  |
| Detachment |  | -.01(-.02, .01) | -.09 | .01 |  | | .01(.00, .02) | | .16 | .01 |  | | .00(-.002, .003) | | -.03 | .001 |  | |  |
| Antagonism |  | -.01(-.02, .003) | -.15 | .01 |  | | .01(-.003, .02) | | .13 | .00 |  | | -.001(-.002, .001) | | -.07 | .001 |  | |  |
| Disinhibition |  | -.02(-.04, -.003)* | -.26 | .01 |  | | .01(-.004, .02) | | .13 | .01 |  | | -.001(-.002, .001) | | -.07 | .001 |  | |  |
| Psychoticism |  | -.01(-.02, .01) | -.10 | .01 |  | | .01(-.004, .02) | | .11 | .01 |  | | .00(-.002, .002) | | -.03 | .001 |  | |  |
| **Social domain** |  |  |  |  |  | |  | |  |  |  | |  | |  |  |  | |  |
| Social problems |  | .01(-.13, .11) | .01 | .06 |  | | .04(-.03, .13) | | .10 | .04 |  | | -.01(-.02, .01) | | -.08 | .01 |  | |  |
| Satisfaction friendships |  | .03(-.03, .10) | .09 | .03 |  | | -.001(-.05, .04) | | .00 | .02 |  | | .003(-.003, .01) | | .09 | .004 |  | |  |
| Satisfaction romantic relations |  | -.02(-.13, .08) | -.04 | .06 |  | | -.02(-.10, .06) | | -.06 | .04 |  | | -.001(-.01, .01) | | -.02 | .01 |  | |  |
| **Work domain** |  |  |  |  |  | |  | |  |  |  | |  | |  |  |  | |  |
| Work satisfaction |  | -.01(-.14, .17) | -.04 | .07 |  | | -.07(-.19, .03) | | -.24 | .05 |  | | -.01(-.03, .01) | | -.25 | .01 |  | |  |
| **Physical domain** |  |  |  |  |  | |  | |  |  |  | |  | |  |  |  | |  |
| Satisfaction general health |  | .02(-.05, .11) | .06 | .04 |  | | -.02(-.08, .04) | | -.06 | .03 |  | | .01(-.003, .01) | | .15 | .01 |  | |  |
| Sleep problems |  | -.01(-.04, .01) | -.09 | .01 |  | | .01(-.01, .03) | | .07 | .01 |  | | .00(-.002, .003) | | -.03 | .002 |  | |  |
| **Self-concept domain** |  |  |  |  |  | |  | |  |  |  | |  | |  |  |  | |  |
| Exploration in breadth |  | -.003(-.03, .03) | -.02 | .02 |  | | .001(-.02, .02) | | .01 | .01 |  | | .002(-.002, .01) | | .13 | .002 |  | |  |
| Ruminative exploration |  | -.02(-.05, .02) | -.09 | .02 |  | | .03(.002, .06) | | .19 | .01 |  | | -.003(-.01, .01) | | -.16 | .003 |  | |  |
| Identification with commitment |  | .04(.01, .06)** | .21 | .01 |  | | -.01(-.03, .01) | | -.09 | .01 |  | | .001(-.003, .003) | | .07 | .002 |  | |  |
| Commitment making |  | .03(-.01, .06) | .14 | .02 |  | | -.01(-.04, .01) | | -.10 | .01 |  | | .003(-.001, .001) | | .14 | .002 |  | |  |
| Exploration in depth |  | -.003(-.03, .04) | .02 | .02 |  | | -.002(-.02, .01) | | -.02 | .01 |  | | .00(-.003, .003) | | .01 | .002 |  | |  |
| Separation from parents |  | -.01(-.03, .01) | -.12 | .01 |  | | .00(-.01, .01) | | .004 | .01 |  | | .00(-.002, .002) | | -.05 | .001 |  | |  |
| Detachment from parents |  | -.002(-.02, .02) | -.02 | .01 |  | | .001(-.01, .01) | | .01 | .01 |  | | .001(-.001, .002) | | .07 | .001 |  | |  |
| Self-efficacy |  | -.003(-.02, .02) | -.03 | .01 |  | | -.002(-.01, .01) | | -.02 | .01 |  | | -.002(-.003, .00) | | -.08 | .001 |  | |  |

Note. *p < .05. ***p* < .01. No significant adjusted p-values for FDR were found, reported are significance values without FDR correction. ^a^ With control variables age of the child and family education.

Online Resource 6

*Multiple Bootstrapped Regression Analyses^a^ with Father Reported Early Childhood Internalizing Problems, Externalizing Problems and*

*Their Interactions and (Mal)Adaptive Functioning in Emerging Adulthood, For Boys.*

|  |  | Internalizing problems | | |  | Externalizing problems | | |  | Internalizing x externalizing problems | | |  |
| --- | --- | --- | --- | --- | --- | --- | --- | --- | --- | --- | --- | --- | --- |
| ***Outcome*** |  | *B(BCa CI)* | β | *SE* *B* |  | *B(BCa CI)* | β | *SE* *B* |  | *B(BCa CI)* | β | *SE* *B* |  |
| **Psychological domain** |  |  |  |  |  |  |  |  |  |  |  |  |  |
| *Internalizing dimension* |  |  |  |  |  |  |  |  |  |  |  |  |  |
| Anxious/depressed behavior |  | .14(-.20, .44) | .09 | .15 |  | .08(-.13, .28) | .07 | .10 |  | -.02(-.06, .003) | -.12 | .02 |  |
| Withdrawn behavior |  | .06(-.08, .19) | .08 | .07 |  | .06(-.03, .14) | .13 | .05 |  | .002(-.02, .02) | .03 | .01 |  |
| Somatic complaints |  | .06(-.04, .19) | .10 | .06 |  | .03(-.03, .10) | .09 | .03 |  | -.01(-.02, .001) | -.19 | .01 |  |
| *Externalizing dimension* |  |  |  |  |  |  |  |  |  |  |  |  |  |
| Aggressive behavior |  | .07(-.09, .22) | .09 | .07 |  | .04(-.06, .13) | .09 | .05 |  | -.02(-.04, -.01)** | -.25 | .01 |  |
| Delinquency |  | .03(-.08, .17) | .05 | .07 |  | .04(-.03, .11) | .12 | .04 |  | .00(-.01, .02) | .00 | .01 |  |
| Intrusive behavior |  | .04(-.07, .16) | .07 | .06 |  | -.01(-.08, .07) | -.02 | .04 |  | -.01(-.02, .01) | -.07 | .01 |  |
| *Other problems* |  |  |  |  |  |  |  |  |  |  |  |  |  |
| Thought problems |  | .04(-.08, .15) | .06 | .06 |  | .06(-.01, .12) | .15 | .04 |  | -.003(-.02, .01) | -.05 | .01 |  |
| Attention problems |  | .11(-.14, .31) | .09 | .12 |  | .07(-.10, .25) | .09 | .08 |  | -.001(-.03, .03) | -.01 | .01 |  |
| *Personality pathology* |  |  |  |  |  |  |  |  |  |  |  |  |  |
| Negative affect |  | .01(-.01, .03) | .10 | .01 |  | .01(-.01, .02) | .12 | .01 |  | -.001(-.004, .00) | -.14 | .001 |  |
| Detachment |  | .01(-.01, .03) | .08 | .01 |  | .01(.002, .02)* | .22 | .01 |  | -.001(-.003, .00) | -.09 | .001 |  |
| Antagonism |  | -.01(-.02, .01) | -.07 | .01 |  | .003(-.01, .02) | .05 | .01 |  | -.002(-.01, .00) | -.21 | .001 |  |
| Disinhibition |  | .01(-.01, .02) | .06 | .01 |  | .004(-.01, .01) | .08 | .004 |  | .00(-.001, .003) | .05 | .001 |  |
| Psychoticism |  | -.004(-.02, .02) | -.05 | .01 |  | .02(.004, .03)** | .25 | .01 |  | .00(-.003, .001) | -.02 | .001 |  |
| **Social domain** |  |  |  |  |  |  |  |  |  |  |  |  |  |
| Social problems |  | .02(-.09, .12) | .04 | .06 |  | .07(.01, .14)* | .18 | .03 |  | .001(-.01, .01) | .01 | .01 |  |
| Satisfaction friendships |  | -.02(-.08, .05) | -.05 | .03 |  | -.05(-.11, .02) | -.21 | .03 |  | -.003(-.01, .01) | -.09 | .01 |  |
| Satisfaction romantic relations |  | .00(-.12, .12) | -.004 | .06 |  | -.06(-.13, .02) | -.15 | .04 |  | .01(-.01, .03) | .10 | .01 |  |
| **Work domain** |  |  |  |  |  |  |  |  |  |  |  |  |  |
| Work satisfaction |  | -.04(-.12, .10) | -.14 | .05 |  | .01(-.05, .05) | .06 | .03 |  | .004(-.01, .02) | .14 | .01 |  |
| **Physical domain** |  |  |  |  |  |  |  |  |  |  |  |  |  |
| Satisfaction general health |  | .02(-.04, .07) | .08 | .03 |  | -.04(-.07, .001)* | -.20 | .02 |  | -.01(-.01, .00) | -.17 | .003 |  |
| Sleep problems |  | -.01(-.03, .01) | -.05 | .01 |  | .02(.003, .03)* | .23 | .01 |  | .00(-.002, .003) | .03 | .001 |  |
| **Self-concept domain** |  |  |  |  |  |  |  |  |  |  |  |  |  |
| Exploration in breadth |  | -.02(-.06, .03) | -.11 | .02 |  | .01(-.01, .03) | .10 | .01 |  | -.003(-.01, .002) | -.19 | .002 |  |
| Ruminative exploration |  | .03(-.01, .06) | .13 | .02 |  | .01(-.02, .04) | .08 | .01 |  | -.003(-.01, .001) | -.14 | .002 |  |
| Identification with commitment |  | -.02(-.05, .01) | -.10 | .02 |  | -.01(-.03, .02) | -.06 | .01 |  | -.001(-.004, .01) | -.04 | .002 |  |
| Commitment making |  | -.02(-.06, .03) | -.08 | .02 |  | -.01(-.04, .01) | -.09 | .01 |  | .00(-.004, .01) | -.002 | .002 |  |
| Exploration in depth |  | .00(-.03, .03) | .001 | .02 |  | .002(-.02, .02) | .02 | .01 |  | -.001(-.01, .004) | -.09 | .002 |  |
| Separation from parents |  | .01(-.01, .02) | .06 | .01 |  | -.002(-.01, .01) | -.04 | .01 |  | .00(-.002, .001) | -.03 | .001 |  |
| Detachment from parents |  | .01(-.01, .02) | .05 | .01 |  | -.01(-.02, .001) | -.15 | .01 |  | .001(-.001, .003) | .05 | .001 |  |
| Self-efficacy |  | -.02(-.03, .003) | -.20 | .01 |  | .002(-.01, .01) | -.02 | .01 |  | .002(-.003, .001) | -.14 | .001 |  |

Note. *p < .05. ***p* < .01. No significant adjusted p-values for FDR were found, reported are significance values without FDR correction. ^a^ With control variables age of the child and family education.
